# Polymorphisms in lncRNA *MIR2052HG* and susceptibility to breast cancer in Chinese population

**DOI:** 10.18632/aging.203686

**Published:** 2021-11-11

**Authors:** Hui Yang, Qiuyu Sun, Feifei Chong, Xiaoru Jiang, Yanli Wang, Kedi Xu, Yuanlin Zou, Linping Xu, Chunhua Song

**Affiliations:** 1 Department of Gastroenterology and Hepatology, Henan Provincial People’s Hospital, People’s Hospital of Zhengzhou University, Jinshui, Zhengzhou 450003, Henan, China; 2 Department of Epidemiology and Statistics, College of Public Health, Zhengzhou University, Zhengzhou 450001, Henan Province, China; 3 Henan Key Laboratory of Tumor Epidemiology, Zhengzhou University, Zhengzhou 450052, Henan Province, China; 4 State Key Laboratory of Esophageal Cancer Prevention and Treatment, Zhengzhou University, Zhengzhou 450052, Henan Province, China; 5 Medical Research Office, The Affiliated Cancer Hospital of Zhengzhou University, Zhengzhou 450003, Henan Province, China

**Keywords:** breast cancer, MIR2052HG, single nucleotide polymorphism, genetic susceptibility, function

## Abstract

Background: Published studies based on pharmacokinetics have explored the relationship between the lncRNA *MIR2052HG* and the prognosis of breast cancer (BC) resistance and recurrence. However, the underlying association of *MIR2052HG* SNPs with BC development remains unclear.

Methods: Combining bioinformatics and databases, SNPs (Single Nucleotide Polymorphisms) in the *MIR2052HG* gene were screened, and SNPs in the lncRNA *MIR2052HG* were selected for genotyping among 504 Chinese Han patients and 505 healthy controls, which were frequency-matched for age (±2 years). Logistic regression analysis was used to explore the association between *MIR2052HG* SNPs and the BC risk. Interactions between the *MIR2052HG* SNPs and reproductive factors were further evaluated using the multifactor dimensionality reduction (MDR) method. qRT–PCR was performed to detect *MIR2052HG* expression in individuals with different genotypes of rs34841297. The target miRNA, miR-4456 of *MIR2052HG* rs34841297 was predicted by websites and confirmed by performing dual luciferase gene reporter assays. CCK-8 and Transwell experiments were designed to explore the effects of miR-4456 on the proliferation, invasion and migration of BC cells.

Results: In this study, nine SNPs were screened. After adjusting for age, menarche age, menopausal status, number of pregnancies, history of abortions, breast feeding history and family history of BC, the results of the logistic regression analysis showed the rs34841297 A/- gene polymorphism was positively correlated with the incidence of BC. Compared with the AA genotype, patients with the A-+-- genotype of rs34841297 at age<50 years, and menarche age<14 years, Premenopausal status, history of abortion, no history of breastfeeding and no family history of tumors in first-degree relatives had an increased risk of BC. MDR results revealed that individuals with rs34841297 - (homozygous deletion) of the A allele who were not menopausal and had no history of breastfeeding had a higher risk of BC. qRT–PCR results revealed that homozygous deletion (1.68±1.37) of the rs34841297 A- genotype resulted in higher *MIR2052HG* expression than the heterozygous deletion genotype (0.95±0.94) and wild AA genotype (0.26±0.12). Binding between *MIR2052HG* and miR-4456 was occurred when rs34841297 carried the AA genotype. Moreover, preliminary functional studies indicated that the overexpression of miR-4456 increased the proliferation, invasion and migration of BC cells.

Conclusion: Our study showed that the *MIR2052HG* gene polymorphism may be related to BC susceptibility, and the *MIR2052HG* rs34841297 A/- genotype may probably affect the proliferation, invasion and migration of BC cells by modulating the interactions with of miR-4456.

## INTRODUCTION

According to the latest global cancer burden data released by the International Agency for Research on Cancer (IARC) of the World Health Organization in 2020 [1], the incidence of breast cancer (BC) in women exceeds that of lung cancer, accounting for 11.7% of all new tumors and becoming the most common cancer in the worldwide. Approximately 685,000 deaths from BC were reported, making it the fifth leading cause of cancer-related death in the world [1], 90% of which were caused by distant metastasis of primary tumor cells [2, 3]. Among women, the BC incidence and mortality rank first among 159 countries in the world, and the relative increase in cancer risk was the largest in low or medium countries/regions (95% increase and 64% increase from 2020, respectively). Although the mortality rate of BC had decreased in 1991-2017 [4], in the past ten years (2008-2017), the rate of decrease in mortality of females with BC has gradually slowed. The aforementioned survey data showed that the previous preventive or treatment measures were not effective, and the high morbidity and mortality rates have become a public health problem that seriously threatens women’s health. Therefore, effective and cost-effective early detection, early diagnosis and individualized treatment of BC have become urgent problems that remain to be solved worldwide.

Current studies have reported many reproductive and environmental factors related to BC [5–7]. Genetic factors are also indispensable for increasing the risk of BC. *BRCA1* and *BRCA2* are currently recognized BC susceptibility genes and are widely measured as predictors of BC risk [8–10]. Recently, with the development of high-throughput sequencing technology, noncoding RNAs have been extensively studied, and lncRNAs, new BC biomarkers, are involved in some biological processes, such as cell proliferation, cell cycle, apoptosis, pluripotency differentiation and maintenance [11], resulting in the occurrence and development of some cancers including BC, liver cancer, and lung cancer, by promoting tumor proliferation, invasion, and metastasis [12–15].

The LncRNA *MIR2052HG*, also known as *FLJ39080* and *LOC441355*, is a long non-coding RNA located on chromosome 8. James N et al. [16] found that the risk of BC recurrence in individuals with homozygous mutant and heterozygous genotypes of the lncRNA *MIR2052HG* was lower than that in the wild type homozygous individuals. In addition, *MIR2052HG* overexpression increases BC cell proliferation and promotes colony formation. Pharmacogenomics studies [17] have shown that as a functional polymorphic gene, *MIR2052HG* might affect the risk of BC recurrence in women treated with aromatase inhibitors. Single Nucleotide Polymorphisms (SNPs) are polymorphisms in DNA sequences caused by variations in single nucleotides at the genome level. According to genome-wide association studies (GWAS) [18], SNPs in lncRNAs are related to susceptibility to many diseases, and SNPs at the key regulatory position of lncRNAs may substantially disrupt their function. Wang L et al. [19] also found that the SNP rs3802201 in *MIR2052HG* is closely related to the recurrence. However, this study was mainly based on pharmacogenomics to explore the relationship between the lncRNA *MIR2052HG* and BC resistance and recurrence. Researchers have not clearly determined whether there is an association between the genetic variants of *MIR2052HG* and BC susceptibility exists.

Therefore, relying on the Han population in Henan, this project screened SNPs in the lncRNA *MIR2052HG* that affect the occurrence of BC and studied the possible molecular mechanism to discover new risk markers for BC. The results might facilitate the early identification and diagnosis of BC in high-risk populations to achieve the purpose of early prevention of BC.

## RESULTS

### Basic characteristics of study subjects

Based on a case-control study, the basic information of 504 patients and 505 healthy controls was presented in Table 1. The results showed that the age at menarche of the case group (14.21±1.70) was higher than that of the control group (13.97±1.75) (*P*=0.030), and ≥2 pregnancies (*OR*:2.049*,* 95*% CI*: 1.592-2.637, *P*<0.001) and the family history of BC (*OR*:1.869 95% *CI*: 1.116-3.130, *P*=0.017) were risk factors for BC. However, a breastfeeding history (*OR*:0.724, 95% *CI*: 0.535-0.980, *P*=0.037) protected against BC.

**Table 1:** Basic characteristics of 504 breast cancer cases and 505 healthy controls.

**Variable**	**Case (%)**	**Control (%)**	***P* ^b^**
	**n=504**	**n=505**	
Age ($\bar{\text{x}}\pm \text{s}$)	48.00 ± 9.85	48.15 ± 9.61	0.806^a^
Age at menarche ($\bar{\text{x}}\pm \text{s}$)	14.21 ± 1.70	13.97 ± 1.75	0.030^a^
Age at menopause ($\bar{\text{x}}\pm \text{s}$)	48.60± 3.90	48.72 ± 3.70	0.760^a^
Menopause statue			
Un-menopause	320 (63.5)	294 (58.2)	
Post-menopause	184 (36.5)	211(41.8)	0.086
Number of pregnancies			
<2	53(10.5)	94(18.6)	
≥2	451 (89.5)	411 (81.4)	<0.001
Number of abortions			
<2	340 (67.5)	345(68.3)	
≥2	164 (32.5)	160 (31.7)	0.771
Breast-feeding history			
No	121 (24.0)	94 (18.6)	
Yes	383 (76.0)	411(81.4)	0.037
Family history			
No	461 (91.5)	481(95.2)	
Yes	43 (8.5)	24 (4.8)	0.017
ER receptor			
Negative	149(30.3)		
Positive	342(69.7)		
PR receptor			
Negative	191(39.1)		
Positive	298(60.9)		
HER-2 receptor			
Negative	136(29.3)		
Positive	328(70.7)		

^a^*t* test.

^b^*χ^2^* test, Two-sided *P<*0.05 is statistically significant.

### Association of *MIR2052HG* SNPs with BC susceptibility

Different genotype models of nine *MIR2052HG* SNPs with BC susceptibility were presented in Table 2. The *MIR2052HG* rs3802201 CG+GG genotype, rs2553716 AC genotype and AC+CC genotype, rs2588297 GT genotype and GT+TT type, rs4259395 AG+GG genotype, rs10957736 TT genotype and CT+TT genotype and rs12546233 AC+CC genotype might reduce the risk of BC, while the rs34841297 deletion mutation increases the risk of breast cancer. Compared with the wild homozygous AA genotype, the SNP rs34841297 --(homozygous deletion) genotype (*OR:* 1.936, *95% CI:* 1.208-3.123) and A-+--genotype (*OR:* 1.704, *95% CI:* 1.087-2.672) resulted in a higher risk in BC.

**Table 2:** Association between nine SNPs and breast cancer susceptibility.

**SNPs**	**Genetic model**	**Genotype**	**Case (n=504, %)**	**Control (n=505, %)**	***P^a^***	**Adjusted *OR* (95%CI)**	***P^b^***
rs3802201	Codominant	CC	257(51)	232(45.9)	0.0545	1	
		CG	218(43.3)	231(45.7)		0.776(0.590,1.022)	0.071
		GG	29(5.8)	42(8.3)		0.642(0.375,1.098)	0.105
	Dominant	CC	257(51.0)	232(45.9)		1	
		CG+GG	247(49.0)	273(54.1)		0.756(0.580,0.986)	0.039
	Recessive	CC+CG	475(94.2)	463(91.7)		1	
		GG	29(5.8)	42(8.3)		0.725(0.431,1.219)	0.225
	Over-Dominant	CC+GG	286(56.7)	274(54.3)		1	
		CG	218(43.3)	231(45.7)		0.822(0.630,1.073)	0.148
rs2553716	Codominant	AA	261(51.8)	231(45.7)	0.1535	1	
		AC	211(41.9)	232(45.9)		0.739(0.560,0.973)	0.031
		CC	32(6.3)	42(8.3)		0.728(0.432,1.228)	0.234
	Dominant	AA	261(51.8)	231(45.7)		1	
		AC+CC	243(48.2)	274(54.3)		0.737(0.565,0.931)	0.024
	Recessive	AA+AC	472(93.7)	463(91.7)		1	
		CC	32(6.3)	42(8.3)		0.841(0.507,1.394)	0.501
	Over-Dominant	AA+CC	293(58.1)	273(54.1)		1	
		AC	211(41.9)	232(45.9)		0.770(0.590,1.006)	0.055
rs4259395	Codominant	AA	186(36.9)	163(32.3)	0.041	1	
		AG	253(50.2)	264(52.3)		0.775(0.579,1.073)	0.086
		GG	65(12.9)	78(15.4)		0.693(0.457,1.453)	0.086
	Dominant	AA	186(36.9)	163(32.3)		1	
		AG+GG	318(63.1)	542(67.7)		0.756(0.573,0.999)	0.049
	Recessive	AA+AG	439(87.1)	427(84.6)		1	
		GG	65(12.9)	78(15.4)		0.807(0.552,1.179)	0.268
	Over-Dominant	AA+GG	251(49.8)	241(47.7)			
		AG	253(50.2)	264(52.3)		0.862(0.662,1.123)	0.272
rs2588297	Codominant	GG	343(68.1)	291(57.6)	0.3605	1	
		GT	147(29.2)	195(38.6)		0.597(0.448,0.794)	0.000
		TT	14(2.8)	19(3.8)		0.799(0.335,1.463)	0.343
	Dominant	GG	343(68.1)	291(57.6)		1	
		GT+TT	161(31.9)	214(42.4)		0.606(0.459,0.798)	0.000
	Recessive	GG+GT	490(97.2)	486(96.2)		1	
		TT	14(2.8)	19(3.8)		0.839(0.404,1.742)	0.637
	Over-Dominant	GG+TT	357(70.8)	310(61.4)		1	
		GT	147(29.2)	195(38.6)		0.608(0.458,0.807)	0.001
rs10957736	Codominant	CC	242(48.0)	211(41.8)	0.2226	1	
		CT	224(44.4)	235(46.5)		0.804(0.608,1.062)	0.124
		TT	38(7.5)	59(11.7)		0.562(0.348,0.907)	0.018
rs10957736	Dominant	CC	242(48.0)	211(41.8)		1	
		CT+TT	262(52.0)	294(58.2)		0.756(0.579,0.986)	0.039
	Recessive	CC+CT	466(92.5)	446(88.3)		1	
		TT	38(7.5)	59(11.7)		0.627(0.397,0.991)	0.046
	Over-Dominant	CC+TT	280(55.6)	270(53.5)		1	
		CT	224(44.4)	235(46.5)		0.889(0.682,1.160)	0.387
rs269183	Codominant	TT	394(78.2)	396(78.4)	0.5904	1	
		CT	106(21.0)	103(20.4)		1.009(0.727,1.399)	0.959
		CC	4(0.8)	6(1.2)		0.580(0.151,2.236)	0.429
	Dominant	TT	394(78.2)	396(78.4)		1	
		CT+CC	110(21.8)	109(21.6)		0.983(0.713,1.355)	0.917
	Recessive	TT+CT	500(99.2)	499(98.8)		1	
		CC	4(0.8)	6(1.2)		0.579(0.151,2.229)	0.427
	Over-Dominant	TT+CC	398(79.0)	402(79.6)		1	
		CT	106(21.0)	103(20.4)		1.015(0.733,1.408)	0.927
rs269198	Codominant	CC	380(75.4)	384(76.0)	0.733	1	
		CA	117(23.2)	114(22.6)		1.036(0.756,1.459)	0.826
		AA	7(1.4)	7(1.4)		0.934(0.309,2.822)	0.903
	Dominant	CC	380(75.4)	384(76.0)		1	
		CA+AA	124(24.6)	121(24.0)		1.029(0.757,1.400)	0.853
	Recessive	CC+CA	497(98.6)	498(98.6)		1	
		AA	7(1.4)	7(1.4)		0.926(0.307,2.793)	0.892
	Over-Dominant	CC+AA	387(76.8)	391(77.4)		1	
		CA	117(23.2)	114(22.6)		1.037(0.758,1.420)	0.820
rs34841297	Codominant	AA	39(7.7)	63(12.5)	0.3673	1	
		A-	223(44.2)	236(46.7)		1.502(0.938,2.405)	0.091
		--	242(48.0)	206(40.8)		1.936(1.208,3.123)	0.006
	Dominant	AA	39(7.7)	63(12.5)		1	
		A-+--	465(92.3)	442(87.5)		1.704(1.087,2.672)	0.020
	Recessive	AA+A-	262(52.0)	299(59.2)		1	
		--	242(48.0)	206(40.8)		1.388(1.062,1.812)	0.016
	Over-Dominant	AA+--	281(55.8)	269(53.3)		1	
		A-	223(44.2)	236(46.7)		0.875(0.671,1.142)	0.326
rs12546233	Codominant	AA	276(54.8)	242(47.9)	0.6976	1	
		AC	195(38.7)	220(43.6)		0.764(0.578,1.006)	0.055
		CC	33(6.5)	43(8.5)		0.664(0.394,1.119)	0.124
	Dominant	AA	276(54.8)	242(47.9)		1	
		AC+CC	228(45.2)	263(52.1)		0.747(0.573,0.973)	0.031
	Recessive	AA+AC	471(93.5)	462(91.5)		1	
		CC	33(6.5)	43(8.5)		0.749(0.451,1.244)	0.264
	Over-Dominant	AA+CC	309(61.3)	285(56.4)		1	

^a^*P* value of Hardy-Weinberg equilibrium in controls.

^b^*P* value of logistic regression analysis with adjusted for age, age at menarche, menopausal status, number of pregnancies and abortions, breast-feeding status, and family history of BC in first degree relative.

### Stratified analysis of the association between *MIR2052HG* SNPs and breast cancer susceptibility

A stratified analysis was conducted to further explore the relationship between the nine SNPs in the *MIR2052HG* gene and BC susceptibility. As shown in Table 3, compared with the rs34841297 AA genotype, the A-+-- genotype of rs34841297 in patients age<50 years (*OR:* 1.998, *95% CI:* 1.072-3.723) with an age at menarche<14 years (*OR:* 2.823, *95% CI:* 1.316-6.005) who were non-menopausal (*OR:* 2.490, *95% CI:* 1.288-4.815), had a history of abortion (*OR:* 2.045, *95% CI:* 1.117-3.744), no history of breastfeeding (*OR:* 3.290, *95% CI:* 1.183-3.070) and had family history of tumors in first-degree relatives (*OR:* 1.905, *95% CI:* 1.183-3.070) had an increased risk of BC. However, the CG+GG genotype of rs3802201, the AC+CC genotype of rs2553716, the AG+GG genotype of rs4259395, the rs2588297 GT+TT genotype, the CT+TT genotype of rs10957736 and the AC+CC genotype of rs12546233 were all found to exert a protective effect on the BC risk.

**Table 3:** Stratification analysis of the nine SNPs and BC susceptibility.

	**rs3802201(CG+GG/CC) OR(95%CI)**	***P^a^***	**rs2553716(AC+CC/AA) OR(95%CI)**	***P^a^***	**rs4259395(AG+GG/AA) OR(95%CI)**	***P^a^***
age						
<50	0.845(0.599,1.193)	0.339	0.822(0.583,1.160)	0.266	0.908(0.638,1.293)	0.749
≥50	0.718(0.466,1.106)	0.133	0.704(0.457,1.085)	0.112	**0.622(0.395,0.979)**	**0.040**
Age at menarche						
<14	0.817(0.532,1.253)	0.354	0.844(0.550,1.294)	0.436	0.813(0.515,1.285)	0.376
≥14	0.736(0.523,1.035)	0.078	0.686(0.488,0.965)	0.031	0.752(0.527,1.072)	0.115
Menopausal state						
Pre-menopausal	0.770(0.542,1.092)	0.142	0.750(0.528,1.064)	0.106	0.822(0.570,1.185)	0.293
Post-menopausal	0.760(0.499,1.158)	0.202	0.749(0.491,1.141)	0.178	0.690(0.930,2.257)	0.101
Age at menopause						
<45	0.945(0.224,3.987)	0.938	0.945(0.224,3.987)	0.938	0.784(0.172,3.571)	0.753
≥45	0.725(0.463,1.135)	0.160	0.709(0.453,1.110)	0.133	0.678(0.424,1.085)	0.105
No. of pregnancies						
<3	0.854(0.572,1.273)	0.438	0.861 (0.577,1.283)	0.462	0.882(0.583,1.333)	0.551
≥3	0.759(0.536,1.074)	0.120	0.702(0.495,0.994)	0.046	0.688(0.473,1.003)	0.052
History of abortion						
no	0.667(0.429,1.036)	0.071	0.665(0.429,1.033)	0.069	0.715(0.448,1.142)	0.160
yes	0.834(0.598,1.164)	0.286	0.789(0.572,1.113)	0.183	0.721(0.507,1.023)	0.067
Breast-feeding						
no	1.773(0.971,3.237)	0.062	1.959(1.072,3.581)	0.029	0.670(0.357,1.260)	0.214
yes	1.230(0.913,1.657)	0.174	1.235(0.917,1.664)	0.165	0.779(0.570,1.064)	0.116
Family history						
no	0.717(0.544,0.944)	0.018	0.691(0.524,0.910)	0.009	0.738(0.554,0.985)	0.039
yes	1.594(0.542,4.691)	0.397	1.838(0.615,5.495)	0.276	0.965(0.291,3.199)	0.953
	**rs2588297(GT+TT/GG) OR(95%CI)**	***P^a^***	**rs10957736(CT+TT/CC) OR(95%CI)**	***P^a^***	**rs269183(CT+CC/TT) OR(95%CI)**	***P^a^***
age						
<50	0.671(0.468,0.963)	0.030	0.934(0.658,1.326)	0.703	1.098(0.730,1.649)	0.654
≥50	0.583(0.373,0.911)	0.018	0.605(0.392,0.932)	0.023	0.795(0.453,1.396)	0.425
Age at menarche						
<14	0.651(0.417,1.019)	0.060	0.915(0.592,1.414)	0.688	0.798(0.481,1.323)	0.381
≥14	0.580(0.407,0.828)	0.003	0.704(0.500,0.990)	0.043	1.203(0.785,1.845)	0.396
Menopausal state						
Pre-menopausal	0.583(0.403,0.844)	0.004	0.840(0.589,1.197)	0.334	1.068(0.707,1.614)	0.755
Post-menopausal	0.679(0.439,1.048)	0.080	0.700(0.459,1.066)	0.097	0.860(0.496,1.492)	0.592
Age at menopause						
<45	1.787(0.416,7.670)	0.435	1.667(0.404,6.880)	0.480	1.226(0.194,7.738)	0.828
≥45	0.599(0.376,0.953)	0.031	0.633(0.404,0.992)	0.046	0.837(0.462,1.516)	0.558
No. of pregnancies						
<3	0.597(0.397,0.912)	0.017	0.783(0.524,1.168)	0.230	1.038(0.633,1.702)	0.884
≥3	0.638(0.446,0.912)	0.014	0.708(0.498,1.005)	0.054	0.866(0.573,1.307)	0.492
Abortion						
no	0.468(0.296,0.739)	0.001	0.619(0.398,0.961)	0.032	1.181(0.698,1.998)	0.534
yes	0.717(0.508,1.013)	0.059	0.795(0.56,1.113)	0.181	0.800(0.528,1212)	0.293
Breast-feeding						
no	0.515(0.271,0.978)	0.043	0.730(0.397,1.342)	0.311	0.990(0.492,1.994)	0.979
yes	0.623(0.457,0.850)	0.003	0.775(0.575,1.045)	0.094	0.989(0.687,1.425)	0.953
Family history						
no	0.551(0.412,0.737)	0.000	0.751(0.570,0.991)	0.043	1.028(0.736,1.437)	0.870
yes	1.877(0.635,5.550)	0.255	0.768(0.256,2.309)	0.639	0.559(0.165,1.896)	0.351
	**rs269198(CA+AA/CC) OR(95%CI)**	***P^a^***	**rs34841297(A-+--/AA) OR(95%CI)**	***P^a^***	**rs12546233(CC+AC/AA) OR(95%CI)**	***P^a^***
age						
<50	1.015(0.909,1.132)	0.795	1.998(1.072,3.723)	0.029	0.809(0.572,1.145)	0.231
≥50	0.864(0.508,1.471)	0.591	1.479(0.737,2.969)	0.271	0.718(0.467,1.10)	0.131
Age at menarche						
<14	0.990(0.614,1.597)	0.967	2.823(1.316,6.055)	0.008	0.881(0.573,1.353)	0.562
≥14	1.133(0.752,1.709)	0.550	1.220(0.679,2.191)	0.506	0.699(0.496,0.984)	0.040
Menopausal state						
Pre-menopausal	1.145(0.767,1.709)	0.508	2.490(1.288,4.815)	0.007	0.720(0.506,1.024)	0.067
Post-menopausal	0.855(0.509,1.438)	0.555	1.063(0.546,2.070)	0.858	0.857(0.563,1.305)	0.472
Age at menopause						
<45	0.725(0.126,4.158)	0.718	0.333(0.027,4.106)	0.391	1.689(0.406,7.026)	0.471
≥45	0.896(0.514,1.562)	0.700	1.214(0.592,2.487)	0.597	0.799(0.510,1.250)	0.325
No. of pregnancies						
<3	1.035(0.646,1.658)	0.888	1.728(0.889,3.358)	0.107	0.688(0.460,1.030)	0.069
≥3	0.918(0.616,1.368)	0.674	1.573(0.861,2.876)	0.141	0.750(0.530,1.060)	0.103
Abortion						
no	1.164(0.707,1.918)	0.550	1.218(0.608,2.438)	0.578	0.517(0.333,0.804)	0.003
yes	0.865(0.580,1.289)	0.475	2.045(1.117,3.744)	0.020	0.889(0.637,1.240)	0.488
Breast-feeding						
no	1.124(0.570,2.216)	0.735	3.290(1.142,9.476)	0.027	0.650(0.357,1.182)	0.158
yes	0.995(0.702,1.410)	0.976	1.504(0.902,2.510)	0.118	0.785(0.583,1.057)	0.111
Family history						
no	1.063(0.771,1.464)	0.710	0.627(0.103,3.820)	0.613	0.737(0.560,0.971)	0.030
yes	0.661(0.199,2.192)	0.498	1.905(1.183,3.070)	0.008	0.943(0.323,2.754)	0.914

*^a^P* values adjusted for age, menarche age, menopausal status, number of pregnancies, number of abortions, history of breast feeding, and family history of breast cancer in first-degree relatives in logistic regression analysis (except for stratification factors).

The distributions of hormone receptor status (ER, PR and HER-2) and molecular subtypes (triple-negative, Her-2 overexpression and luminal) of patients with BC presenting different SNP genotypes are shown in Supplementary Tables 4 and 5, respectively. The rs269183 T>C variant was associated with Her-2 receptor positive and Her-2 overexpressing BC. The rs269198 C>A variant reduces the risk of Her-2-overexpressing BC. The GG genotype of rs4259395 was related to the PR receptor-positive BC, and the TT genotype of rs2588297 was related to the Luminal-type BC. SNPs rs3802201 C>G and rs2553716 A>C were both associated with PR positivity and luminal BC. In addition, the rs12546233 AC+CC genotype was associated with triple-negative BC and the AA genotype was associated with Her-2-overexpression BC.

### False positive report probability (FPRP)

FPRP analysis [20] was used to evaluate the reliability of the positive results for *MIR2052HG* SNPs associated with BC susceptibility. As presented in Supplementary Table 6, when the critical value of FPRP was set to 0.5 and prior probability was 0.25, the FPRP values of all positive results for SNPs rs2553716, rs269183, rs269198, and rs12546233 were lower than the critical value. A possible association between the SNPs and the risk of BC was observed, which was worthy of further research and verification.

### Haplotype analysis

Haplotype analysis was used to test the combined effect of *MIR2052HG* SNPs (Supplementary Table 7). Haplotype C_rs3802201_A_rs2553716_A_rs4259395_G_rs2588297_C_rs10957736_T_rs269183_C_rs269198_ W_rs34841297_A_rs12546233_ was the highest frequency haplotype, and individuals with this haplotype have an increased risk of BC (*OR:* 1.203, 95% *CI*: 1.001-1.445), while Haplotype G_rs3802201_C_rs2553716_G_rs4259395_T_rs2588297_ T_rs10957736_ T_rs269183_C_rs269198_M_rs34841297_ A_rs12546233_ and haplotype G_rs3802201_C_rs2553716_G_rs4259395_T_rs2588297_T_rs10957736_T_rs269183_C_rs269198_M_rs34841297_C_rs12546233_ were associated with a low risk of BC (*OR:* 0.258, 95 %*CI*: 0.073-0.908; *OR:* 0.698, 95% *CI*: 0.549-0.887, respectively).

### Multifactor dimensionality reduction

MDR software (multifactor dimensionality reduction 3.0.2) was used to analyze the interaction of genes and reproductive factors. As shown in Table 4, there was an interaction with the rs34841297 - (homozygous deletion) of A genotype, non-menopausal status, and no history of breastfeeding was observed, furthermore, the interaction model revealed a higher risk of BC (*OR:* 1.771, 95% *CI*: 1.367-2.941, *P*<0.001).

**Table 4:** Interaction results between *MIR2052HG* SNPs and reproductive factors.

**Model**	**TBA^a^**	**CVC^b^**	***χ2***	***P***	***OR(95%CI)***
Pre-menopausal, no breast-feeding	0.4966	3/10	10.061	0.002	1.543(1.180,2.018)
Pre-menopausal, no breast-feeding, rs34841297	0.5313	7/10	18.883	<0.001	1.771(1.367,2.941)

^a^Testing balance accuracy.

^b^Cross-validation consistency.

### Real-time PCR results

**Figure 1:**
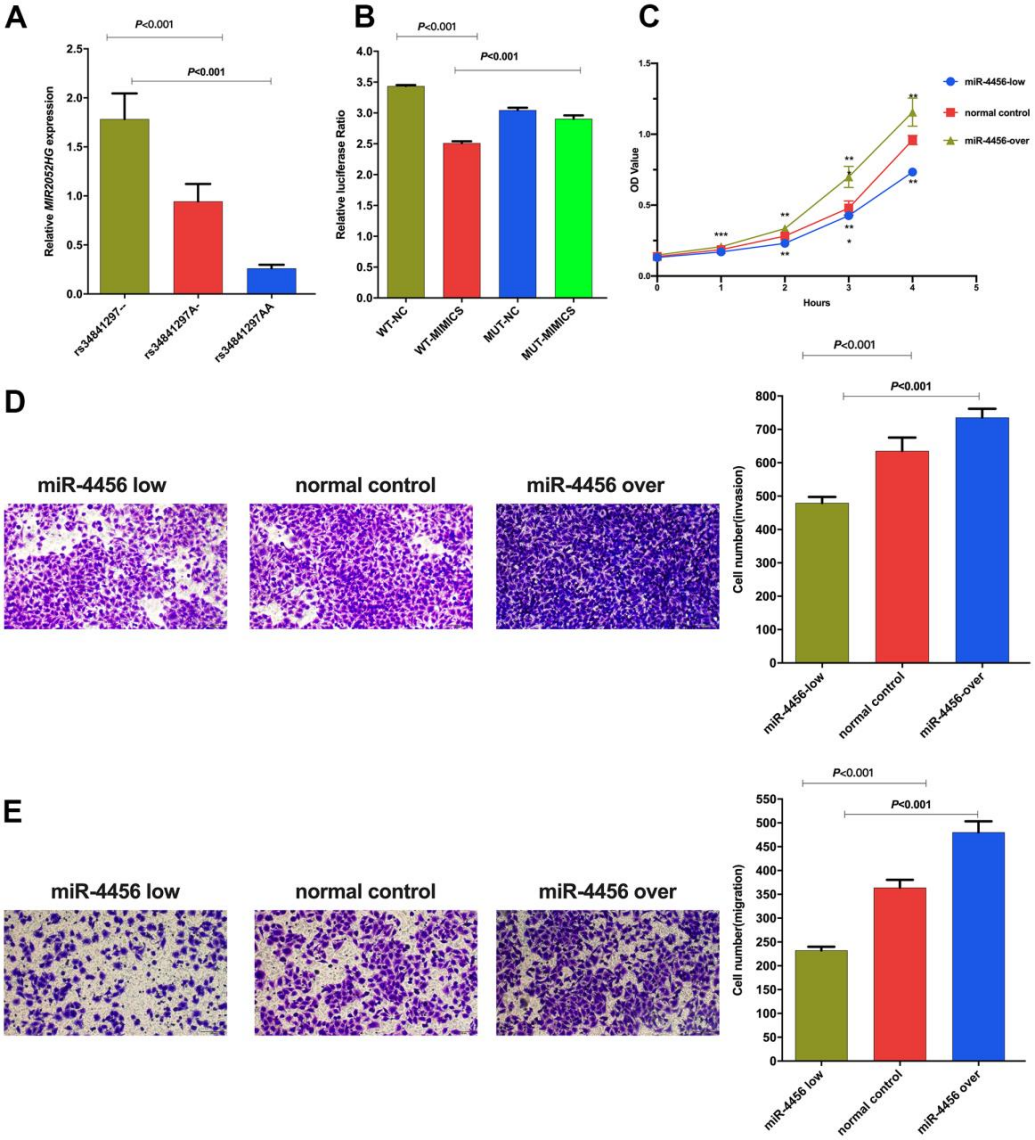
(**A**) Relative expression of LncRNA *MIR2052HG* in different genotypes of rs34841297. (**B**) The relative fluorescence value of different experimental groups in the dual luciferase report experiment. WT-NC: rs34841297 wild plasmids-Normal Control mimics, WT-MIMIC: rs34841297 wild plasmids-miR-4456 mimics, MUT-NC: rs34841297 mutant plasmids-Normal Control mimics, MUT-MIMIC: rs34841297 mutant plasmids-miR-4456 mimics. (**C**) The effect of miR-4456 combined with *MIR2052HG* on the proliferation of breast cancer cells (0-96h). ^*^represents *P*<0.05; ^**^represents *P*<0.01; ^***^represents *P*<0.001. (**D**) Transwell experiment explores the effect of miR-4456 combined with *MIR2052HG* on the invasion ability of MDA-MB-231 cells. (**E**) Transwell experiment explores the effect of miR-4456 combined with *MIR2052HG* on the migration ability of MDA-MB-231 cells.

The *MIR2052HG* expression levels in individuals with the rs34841297 --, A- and AA genotypes were shown in Figure 1A. The relative expression in individuals with the homozygous deletion genotype (1.68±1.37) was significantly higher than that in individuals with the heterozygous deletion genotype (0.94±0.95) (*P*=0.011) and AA genotype (0.26±0.12) (*P*<0.001). In addition, the relative expression of *MIR2052HG* in individuals with a homozygous deletion of rs34841297 was significantly higher than that of individuals with the AA genotype (*P*=0.001).

### Dual-luciferase reporter assays

A dual-luciferase reporter assay was performed in 293T cells to determine the biological association between rs34841297 and miR-4456. As shown in Figure 1B, the relative luciferase activity of the rs34841297 W-NC group was significantly higher than rs34841297 W-miR-4456 group (*P*<0.001), suggesting an interaction between the rs34841297 wild genotype plasmid vector and miR-4456. Meanwhile, the relative luciferase activity of the rs34841297 W-miR-4456 group was lower than the rs34841297 MUT-miR-4456 group (*P*<0.001), showing that the interaction between the plasmid vector of *MIR2052HG* and miR-4456 disappeared due to the deletion of the rs34841297 A allele.

### The effect of miR-4456 combined with *MIR2052HG* on cell proliferation, invasion and migration

The relative expression of *MIR2052HG* and miR-4456 in MDA-MB-231 cells (2.02±0.34, 3.95±0.65) were both higher than that in MCF10A cells (0.92±0.08, 1.17±0.79), and the differences were statistically significant (Supplementary Figure 1).

qRT–PCR was performed to detect the relative expression of miR-4456 in BC cells (MDA-MB-231) stably transfected with three lentiviruses. The results of the CCK-8 test are shown in Figure 1C. In the MDA-MB-231 cells, the OD values for the miR-4456 low-expression group at 24 h (*P*<0.001), 48 h (*P*=0.001), 72 h (P<0.001) and 96 h (*P*=0.002) were lower than those of the NC group, and the OD values for the miR-4456 overexpression group at 24 h (*P*<0.001), 48 h (*P*=0.005), 72 h (*P*<0.001) and 96 h (*P*=0.001) were higher than those of the NC group. Then, the number of cells passing through the Matrigel-covered Transwell chamber was observed to determine the ability of miR-4456 to modulate the invasion of MDA-MB-231 cells. The results presented in Figure 1D showed that the number of invading MDA-MB-231 cells in the miR-4456 low-expression group was lower than that in the NC group (*P*<0.001), and the number of invading MDA-MB-231 cells in the miR-4456 overexpression group was higher than that in the NC group (*P*=0.002). Finally, Transwell experiments were performed to explore the migration ability of BC cells with different miR-4456 expression levels. As presented in Figure 1E, the number of migrating MDA-MB-231 cells in the miR-4456 low-expression group MDA-MB-231 was lower than that in the NC group (*P*=0.004), and the number of migrating MDA-MB-231 cells in the miR-4456 overexpression group MDA-MB-231 was higher than that in the NC group (*P*=0.022).

## DISCUSSION

In the present study, six SNPs rs3802201 (C>G), rs2553716 (A>C), rs4259395 (A>G), rs2588297 (G>T), rs10957736 (C>T) and rs12546233 (A>C), reduced the risk of BC. Among them, the rs3802201 (C>G) mutation reduces the risk of breast cancer, which corresponds with the study by Wang L et al. [19]. However, the rs34841297 gene polymorphism was positively correlated with the incidence of BC, and rs34841297 A gene deletion might increase the risk of BC. Furthermore, the associations between nine *MIR2052HG* SNPs and the BC receptor status (ER, PR, Her-2) were analyzed. Estrogen receptor (ER) can regulate normal breast epithelial cells and breast gland proliferation of cancer cells [21, 22]. Progesterone receptor (PR) was a member of the nuclear receptor superfamily of transcription factors that has the biological function of promoting functional recovery and reducing the volume of BC lesions [23]. Human epidermal growth factor receptor 2 (Her-2), as a marker for predicting the prognosis of BC, was regarded as the key to evaluating the efficacy of targeted drugs [16]. Our results indicated that rs3802201, rs2553716 and rs4259395 may exert a protective effect on BC by affecting PR receptor status. The CT+CC genotype of rs269183 was related to Her-2 receptor status and may affect the prognosis of BC. MDR model results showed that with rs34841297, homozygous deletion of the A gene, a non-menopausal status and no history of breastfeeding resulted in a higher risk of BC, which might lead to an increased risk of BC.

*MIR2052HG* downregulation expression was reported to reduce ERα-positive BC cell growth [24]. Genetic variations in *MIR2052HG* were associated with the BC-free interval in the MA.27 trial (ClinicalTrials.gov number NCT00066573), and the variant SNPs were associated with increased *MIR2052HG* expression due to increased ERα binding to EREs [17, 25]. What’s more, researchers have discovered that SNPs could mediate the occurrence of cancer by affecting the secondary structure and expression of lncRNAs and the biological effects mediated by the interactions between lncRNAs and miRNAs [26–28]. Here, we performed qRT–PCR to explore the total expression of *MIR2052HG* expression in individuals with different genotypes of rs34841297. A dual-luciferase reporter gene experiment was conducted to identify whether the rs34841297 A/- deletion mutation affects the binding ability of *MIR2052HG* to miR-4456. An interaction was observed between *MIR2052HG* and miR-4456 mimics when rs34841297 carried wild-type A allele, and the interaction disappeared with the deletion of the A allele, consistent with the results predicted by the LncRNASNP2 and DINAN websites. This result suggested that the rs34841297 polymorphism might affect the *MIR2052HG* and miR-4456 interaction.

MiRNAs are small noncoding RNAs of approximately 22 nucleotides in length that perform posttranscriptional regulatory functions by binding to specific sites on the target transcript [29]. miR-4456 is a tiny noncoding RNA located on chromosome 5 that putatively influences oxytocin signaling [30]. To date, no studies have reported the relationship between miR-4456 and cancer. Our study proposed the hypothesis that the effect of the lncRNA *MIR2052HG* on BC occurrence and development is based on the interaction of *MIR2052HG* and miR-4456 mediated by rs34841297. CCK-8, Transwell and scratch wound experiments were performed using MDA-MB-231 cells. Compared with the normal control group, the proliferation ability of MDA-MB-231 cells with high miR-4456 expression was significantly increased. The Transwell assays using MDA-MB-231 cells found that compared with the normal control group, the ability of cells with high miR-4456 expression to migrate through the chamber and invade the Matrigel-coated Transwell chamber was significantly increased. Based on these experiments, we preliminarily suggested that the lncRNA *MIR2052HG* might mediate the binding of miR-4456 through rs34841297 to affect the proliferation, invasion and metastasis of BC.

This study is the first to explore the association between genetic variants in the lncRNA *MIR2052HG* and BC susceptibility. This study has several advantages. First, the patients included in our study were all newly diagnosed and the controls were selected according to the frequency matching, which might reduce the selection bias in the study. Second, SNPscan high-throughput typing technology was used to SNP typing, making the results more accurate and credible than traditional restriction fragment length polymorphism (PCR-RFLP) typing technology. Finally, genotyping of all SNPs was performed on 10% of randomly selected samples for sequencing verification. Additionally, all cell function experiments were repeated more than three times, which improved the authenticity and reliability of the study results. Nevertheless, this study still has some limitations. All the subjects included in this study were of the Chinese Han population, and further studies of other populations should be performed to verify our results. In addition, the mechanism by which *MIR2052HG* SNPs modulate BC must be further explored *in vivo*.

## CONCLUSIONS

In conclusion, the study reveals the association between the *MIR2052HG* gene polymorphism and the occurrence of BC. The *MIR2052HG* rs34841297 A/-- variant may affect the binding of miR-4456 to *MIR2052HG* and subsequently alter proliferation, invasion and migration of BC cells by regulating the expression of miR-4456 expression, which provides a baseline information for screening high-risk population populations for BC and formulating individualized preventive measures.

## MATERIALS AND METHODS

### Research sample

Based on a case-control study design, 504 new BC samples that were pathologically confirmed were collected from March 2016 to December 2018. All patients were recruited from the top three hospitals in Henan Province. Through frequency matching to patients by age (±2 years), 505 healthy control samples were obtained from the biological sample bank of Henan Key Laboratory of Oncology Epidemiology. The basic information and clinical characteristics of the patients included age, age at menarche, menopausal status, menopausal age, number of pregnancies, number of abortions, history of breastfeeding and family history of BC. Moreover, information on patients’ hormone receptor status including estrogen receptor (ER), progesterone receptor (PR) and human epidermal growth factor receptor-2 (HER-2) was also acquired. The study was approved by the Ethics Review Committee of the Ethics Committee of Medical and Health Research of Zhengzhou University, Zhengzhou, Henan, China.

### DNA extraction, SNP selection and genotyping

Sample DNA was extracted using a DNA Extraction Kit (Shanghai Laifeng Biotechnology Co., Ltd.) according to the manufacturer’s instructions and stored at -80° C until use. The *MIR2052HG* SNPs were screened using Ensembl38, Ensembl37, NCBI 1000 Genomes and Haploview software, with a minor allele frequency (MAF)>0.05 in Chinese Han population. Finally, 9 SNPs in functional regulatory regions SNPs and tag SNPs were determined, and the basic information was presented in Supplementary Table 1. RNAfold was used to predict the secondary structure of the significant SNPs. LncRNASNP2 and DIANA were used to predict the binding capacity of miRNAs that SNPs might affect (Supplementary Table 2). Combined with the Δ Energy, correlation with BC, and site prediction intersection results, miR-4456 was selected for further functional research. All SNPs were genotyped with SNPscan™ multiple SNP typing kit.

### Quantitative real-time PCR (qRT–PCR)

Plasma RNA was extracted from 72 randomly selected healthy controls with TRIzol reagent, DNA was removed and RNA was reverse transcribed into cDNA. The relative expression of *MIR2052HG* in individuals carrying the rs34841297 polymorphism was detected using qRT–PCR with SYBR-green among individuals with different genotypes of rs34841297, and GAPDH served as the endogenous control. All samples were analyzed in triplicate, and the relative expression was calculated using the method of 2^-ΔCt^ method. The sequences of primers used in this study were listed in Supplementary Table 3.

### Dual-luciferase report assay

According to LncRNASNP2, carried with wild-type *MIR2052HG*-rs34841297 might gain a binding site for miR-4456, and the dual-luciferase assay verified the biological association between rs34841297 and miR-4456. Following the construction of the *MIR2052HG* rs34841297 wild-type and mutant pmirGLO plasmids, cotransfected HEK 293T cells with miR-4456 mimic or normal control (NC) mimics by using the riboFECT™ CP kit. Firefly fluorescence activity and Renilla fluorescence activity in each group were detected 72 hours after transfection, and relative luciferase activity was calculated based on firefly/Renilla fluorescence.

### Cytological function experiment

qRT–PCR experiments first detected the relative expression of *MIR2052HG* and miR-4456 in MDA-MB-231 cells and MCF10A cells. The relative expression was calculated using the method of 2-ΔΔCt method. The sequences of *MIR2052HG* primers used in this study were listed in Supplementary Table 3. And the sequences of miR-4456 and U6 internal reference primers are shown in Supplementary Table 8.

BC cells (MDA-MB-231) stably transfected with low-expression, overexpression and negative control (NC) lentiviral vectors carrying miR-4456 were established. Then, the stably transfected cell line was used for CCK-8 experiments and Transwell experiments to detect the proliferation, invasion and migration ability of BC cells with different miR-4456 expression levels.

### Statistical analysis

Unconditional logistic regression analysis was used to explore BC-related *MIR2052HG* SNPs and adjusted for age, menarche age, menopausal status, number of pregnancies, number of abortions, breastfeeding history, and family history. SHEsis online software was applied to conduct the haplotype analysis of *MIR2052HG*. Multifactor dimensionality reduction (MDR) was used to analyze the interaction between genes and the environment. Independent *t* tests were applied to compare the relative expression of the lncRNA *MIR2052HG* with different rs34841297 genotypes, and the false-positive report probability (FPRP) [20] analysis was conducted to verify the positive results and the cut-off value to ensure the reliability and accuracy of the positive results. A *t* test was used to compare the OD values from the CCK8 experiment among groups with low expression of miR-4456 and the high expression of miR-4456 and the NC group. Independent *t* tests were used to analyze the accurate counts of stained cells in the two groups obtained in Transwell experiments. Differences in invasion and migration capabilities were calculated using *t* tests. All data analysis and cell number statistical analyses in this study were performed using SPSS 21.0(t-test, χ2 test and unconditional logistic regression model analysis), ImageJ (calculate scratch healing area, Transwell migration and invasion cell count), and GraphPad Prism 7.0(plot the calculation results). Two-sided *P*<0.05 was statistically significant.

### Informed consent

Informed consent was obtained from all individual participants included in the study.

### Ethical approval

All procedures performed in studies involving human participants were in accordance with the ethical standards of the institutional and/or national research committee and with the 1964 Helsinki declaration and its later amendments or comparable ethical standards.

### Ethical responsibilities of authors

The manuscript has not been submitted to more than one journal for simultaneous consideration. The manuscript has not been published previously (partly or in full).

## Supplementary Material

Supplementary Figure 1

Supplementary Tables
