# Supplementary material for: Polymorphisms in lncRNA MIR2052HG and susceptibility to breast cancer in Chinese population
Source: Aging (Albany NY). 2021 Nov 11;13(21):24360–78. doi: 10.18632/aging.203686 (PMC8610136; doi:10.18632/aging.203686)
Supplement: Supplementary Figure 1 [file aging-13-203686-s001.pdf]

SUPPLEMENTARY FIGURE

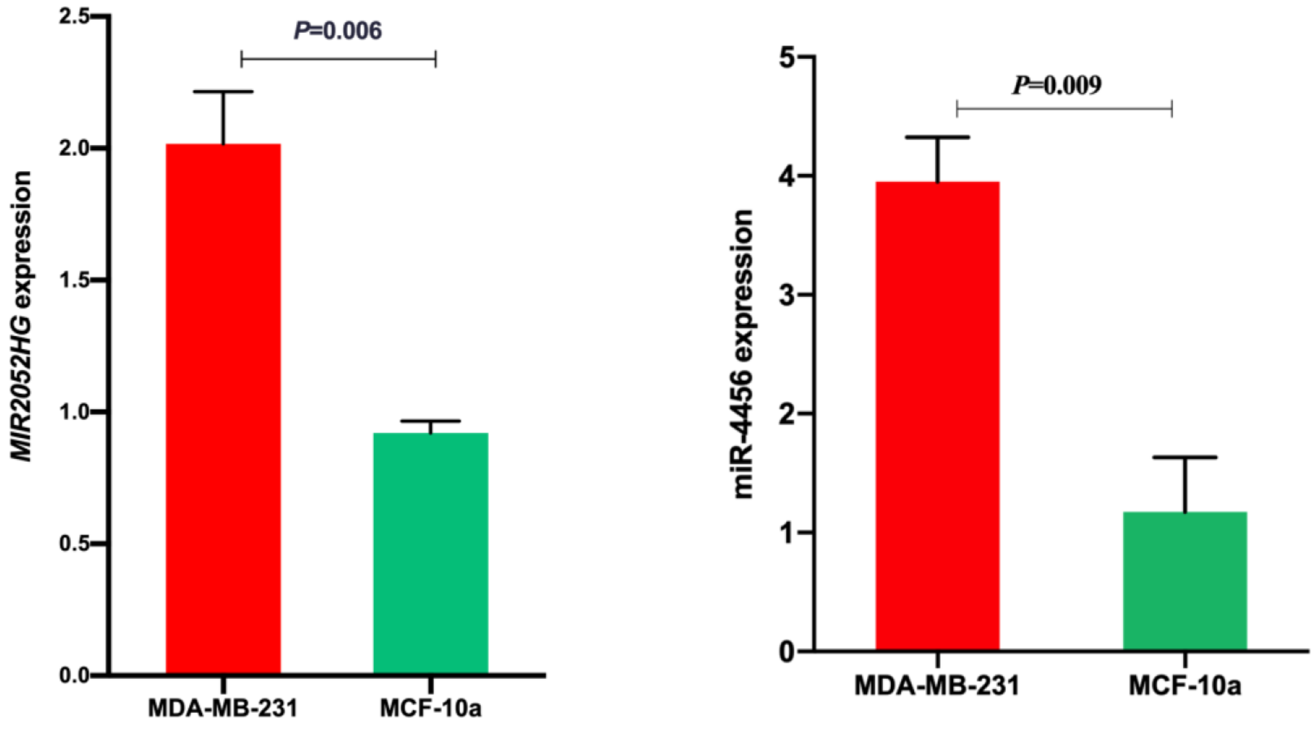

Supplementary Figure 1. Relative expression of *MIR2052HG* and miR-4456 in MDA-MB-231 and MCF-10A cells.
