# Supplementary material for: Polymorphisms in lncRNA MIR2052HG and susceptibility to breast cancer in Chinese population
Source: Aging (Albany NY). 2021 Nov 11;13(21):24360–78. doi: 10.18632/aging.203686 (PMC8610136; doi:10.18632/aging.203686)
Supplement: Supplementary Tables [file aging-13-203686-s002.pdf]

SUPPLEMENTARY TABLES

Supplementary Table 1. Basic information of nine *MIR2052HG* SNPs.

SNP	Position	Ref Allele	%	Alt Allele	%
rs3802201	8:75678754	C	0.705	G	0.295
rs2553716	8:75696873	A	0.705	C	0.295
rs4259395	8:75702373	A	0.602	G	0.398
rs2588297	8:75732970	G	0.797	T	0.203
rs10957736	8:75750323	C	0.679	T	0.321
rs269183	8:75758791	T	0.890	C	0.110
rs269198	8:74702379	C	0.874	A	0.126
rs34841297	8:74752460	-	0.676	A	0.324
rs12546233	8:74758311%	A	0.721	C	0.279

Supplementary Table 2. Biological function prediction of *MIR2052HG* functional SNPs.

LncRNA	SNP	Ref/alt	ΔEnergy (kCal/Mol)	miRNA	Energy (kCal/Mol)	Effect
MIR2052HG	rs12546233	A/C	3	has-miR-4659b-3p	-0.20	loss
				has-mir-4659a-3p	-0.20	loss
	rs269198	G/T	0.3	has-miR-141-5p	-3.70	gain
				has-miR-3126-3p	-1.60	gain
				has-miR-3686	-0.40	gain
				has-miR-452-3p	-1.00	gain
				has-miR-1207-3p	0.00	loss
				has-miR-2115-5p	-4.10	loss
				has-miR-1537-5p	-3.60	loss
	rs34841297	-/A	2.4	hsa-miR-185-3p	-4.90	gain
				hsa-miR-4498	-3.20	gain
				hsa-miR-5001-5p	-4.70	gain
				hsa-miR-4492	-4.00	gain
				hsa-miR-1298-3p	0.00	gain
				hsa-miR-762	-7.20	gain
				**has-miR-4456**	**-0.70**	**loss**
				has-miR-6842-3p	-3.50	loss

Supplementary Table 3. Sequence of MIR2052HG primers of qRT-PCR.

Gene	Sequence of primers[a]
MIR2052HG	F: ATCAGCGAGATTCCGTGGG
	R: GAAACTGCCTCATCAGACATAAAAG
GAPDH	F: CGGAGTCAACGGATTTGGTCGTAT
	R: AGCCTTCTCCATGGTGGTGAAGAC

[a] qRT-PCR method was carried out in plasma to detect the relative MIR2052HG expression in AA genotype, A- genotype and -- genotype of rs34841297. Meanwhile the qRT-PCR was also performed to explore the relative *MIR2052HG* expression in MDA-MB-231cells and MCF10A cells.

Supplementary Table 4. The Associations between *MIR2052HG* SNPs and ER, PR and HER-2 status of breast cancer patients.

Genotype	ER		P^a	OR(95%CI)	PR		P^a	OR(95%CI)	HER-2		P^a	OR(95%CI)
	Negative (n=149)	Positive (n=342)			Negative (n=191)	Positive (n=298)			Negative (n=138)	Positive (n=329)		
rs3802201												
CC	80	169		1	106	142		1	66	168		1
CG	63	150	0.430	1.178(0.785,1.768)	77	135	0.109	1.371(0.932,2.018)	60	144	0.877	0.967(0.633,1.478)
GG	6	23	0.090	2.303(0.877,6.050)	8	21	0.048	2.424(1.006,5.837)	12	17	0.311	0.656(0.290,1.484)
CG+GG	69	173	0.248	1.263(0.850,1.877)	85	156	0.049	1.458(1.001,2.124)	72	161	0.688	0.920(0.611,1.385)
rs2553716												
AA	82	170		1	107	144		1	68	169		1
AC	58	149	0.212	1.300(0.851,1.962)	74	132	0.093	1.397(0.946,2.061)	54	144	0.595	1.124(0.730,1.730)
CC	9	23	0.333	1.514(0.653,3.510)	10	22	0.103	1.966(0.872,4.433)	16	16	0.066	0.485(0.224,1.049)
AC+CC	67	172	0.165	1.326(0.891,1.974)	84	154	0.050	1.457(1.000,2.124)	70	160	0.959	0.989(0.656,1.491)
rs4259395												
AA	56	124		1	74	106		1	53	116		1
AG	75	172	0.581	1.128(0.736,1.728)	98	147	0.633	1.103(0.737,1.650)	63	171	0.226	1.320(0.842,2.068)
GG	18	46	0.364	1.350(0.707,2.580)	19	45	0.046	1.905(1.012,3.585)	22	42	0.933	0.973(0.517,1.833)
AG+GG	93	218	0.455	1.169(0.776,1.760)	117	192	0.302	1.226(0.833,1.805)	85	213	0.335	1.233(0.805,1.888)
rs2588297												
GG	107	226		1	134	198		1	92	223		1
GT	40	104	0.213	1.327(0.850,2.070)	54	89	0.445	1.176(0.776,1.783)	43	95	0.906	0.973(0.622,1.524)
TT	2	12	0.112	3.484(0.476,16.247)	3	11	0.081	3.249(0.867,12.183)	3	11	0.401	1.772(0.467,6.727)
GT+TT	42	116	0.112	1.424(0.921,2.200)	57	100	0.239	1.276(0.851,1.913)	46	106	0.920	1.023(0.661,1.583)
rs10957736												
CC	68	166		1	90	144		1	70	152		1
CT	72	148	0.633	0.905(0.601,1.363)	86	132	0.874	0.969(0.656,1.431)	61	147	0.361	1.220(0.796,1.869)
TT	9	28	0.398	1.423(0.627,3.229)	15	22	0.798	1.099(0.533,2.269)	7	30	0.098	2.132(0.869,5.230)
CT+TT	81	176	0.856	0.964 (0.649,1.431)	101	154	0.949	0.988(0.680,1.436)	68	177	0.192	1.315(0.871,1.986)
rs269183												
TT	110	274		1	145	238		1	116	249		1
CT	38	65	0.155	0.713(0.448,1.136)	44	58	0.378	0.816(0.519,1.282)	22	76	0.057	1.683(0.985,2.875)
CC	1	3	0.839	1.271(0.126,12.867)	2	2	0.696	0,667(0.088,5.062)	0	4	NC	NC
CT+CC	39	68	0.175	0.728(0.460,1.152)	46	60	0.353	0.810(0.519,1.264)	22	80	0.039	1.755(1.030,2.991)
rs269198												
CC	105	265		1	139	230		1	109	243		1
CA	42	72	0.130	0.707(0.451,1.108)	48	65	0.414	0.834(0.539,1.290)	29	79	0.298	1.300(0.793,2.131)
AA	2	5	0.967	1.036(0.192,5.581)	4	3	0.368	0.494(0.106,2.297)	0	7	NC	NC
CA+AA	44	77	0.147	0.722(0.465,1.121)	52	68	0.326	0.808(0.528,1.237)	29	86	0.175	1.403(0.860,2.290)
rs34841297												
AA	10	28		1	15	23		1	7	31		1
A-	68	151	0.583	0.799(0.360,1.776)	85	132	0.728	0.879(0.424,1.820)	62	146	0.199	0.554(0.225,1.366)
--	71	163	0.508	0.766(0.348,1.686)	91	143	0.746	0.888(0.434,1.820)	69	152	0.106	0.478(0.196,1.169)
A-+--	139	314	0.528	0.781(0.363,1.681)	176	275	0.728	0.884(0.441,1.772)	131	298	0.132	0.512(0.214,1.225)
rs12546233												
AA	80	187		1	101	166		1	79	174		1
AC	64	128	0.590	0.894(0.593,1.346)	79	111	0.411	0.848(0.572,1.256)	52	130	0.315	1.248(0.810,1.920)
CC	5	27	0.078	2.459(0.904,6.694)	11	21	0.471	1.335(0.609,2.928)	7	25	0.253	1.695(0.686,4.183)
AC+CC	69	155	0.955	1.012(0.681,1.502)	90	132	0.613	0.908(0.624,1.321)	59	155	0.211	1.302(0.861,1.969)

$^a P$ values adjusted for age, menarche age, menopausal status, number of pregnancies, number of abortions, history of breast feeding, and family history of breast cancer in first-degree relatives in logistic regression analysis.

Supplementary Table 5. The Associations between *MIR2052HG* SNPs and luminal breast cancer, Her-2 over-expression breast cancer and triple-negative breast cancer.

Genotype	Triple-negative		P^a	OR(95%CI)	Her-2 over-expression		P^a	OR(95%CI)	Luminal		P^a	OR(95%CI)
	no (n=418)	yes (n=49)			no (n=390)	yes (n=77)			no (n=195)	yes (n=272)		
rs3802201												
CC	211	23		1	192	42		1	107	127		1
CG	182	22	0.906	1.039(0.550,1.965)	171	33	0.521	0.847(0.509,1.407)	80	124	0.116	1.370(0.925,2.028)
GG	25	4	0.869	1.105(0.336,3.640)	27	2	0.105	0.291(0.065,1.296)	8	21	0.026	2.730(1.127,6.609)
CG+GG	207	26	0.881	1.048(0.566,1.939)	198	35	0.305	0.770(0.467,1.269)	88	145	0.045	1.478(1.009,2.146)
rs2553716												
AA	213	24		1	195	42		1	109	128		1
AC	180	18	0.523	0.805(0.414,1.565)	165	33	0.691	0.902(0.543,1.500)	76	122	0.069	1.443(0.972,2.144)
CC	25	7	0.204	1.895(0.706,5.085)	30	2	0.089	0.274(0.062,1.215)	10	22	0.054	2.233(0.985,5.061)
AC+CC	205	25	0.871	0.950(0.512,1.761)	195	35	0.395	0.805(0.488,1.327)	86	144	0.030	1.526(1.041,2.237)
rs4259395												
AA	151	18		1	141	28		1	74	95		1
AG	211	23	0.587	0.829(0.422,1.630)	194	40	0.956	0.985(0.574,1.689)	101	133	0.643	1.102(0.730,1.666)
GG	56	8	0.896	0.940(0.369,2.390)	55	9	0.459	0.731(0.319,1.676)	20	44	0.031	2.006(1.065,3.778)
AG+GG	267	31	0.629	0.854(0.450,1.619)	249	49	0.777	0.928(0.552,1.559)	121	177	0.281	1.243(0.837,1.846)
rs2588297												
GG	280	35		1	260	55		1	137	178		1
GT	124	14	0.434	0.760(0.382,1.512)	118	20	0.404	0.786(0.446,1.383)	55	83	0.340	1.228(0.806,1.871)
TT	14	0	1.000	0.000	12	2	0.585	0.650(0.138,3.057)	3	11	0.052	3.716(0.987,13.996)
GT+TT	138	14	0.266	0.677(0.341,1.346)	130	22	0.354	0.772(0.446,1.335)	58	94	0.158	1.343(0.892, 2.024)
rs10957736												
CC	195	27		1	190	32		1	91	131		1
CT	187	21	0.220	0.673(0.357,1.267)	171	37	0.334	1.297(0.765,2.199)	89	119	0.852	0.963(0.647,1.433)
TT	36	1	0.092	0.170(0.022,1.339)	29	8	0.432	1.429(0.587,3.434)	15	22	0.561	1.242(0.598,2.578)
CT+TT	223	22	0.098	0.592 (0.318,1.101)	200	45	0.283	1.319(0.796,2.184)	104	141	0.991	1.002(0.685,1.466)
rs269183												
TT	323	42		1	312	53		1	147	218		1
CT	91	7	0.139	0.524(0.223,1.233)	75	23	0.039	1.802(1.031,3.150)	46	52	0.334	0.797(0.504,1.262)
CC	4	0	1.000	0.000	3	1	0.742	1.477(0.145,15.059)	2	2	0.781	0.749(0.098,5.740)
CT+CC	95	7	0.112	0.510(0.217,1.198)	78	24	0.039	1.787(1.031,3.097)	48	54	0.321	0.795(0.506,1.250)
rs269198												
CC	313	39		1	301	51		1	141	211		1
CA	98	10	0.377	0.712(0.335,1.514)	84	24	0.056	1.710(0.987,2.962)	50	58	0.355	0.811(0.520,1.265)
AA	7	0	1.000	0.000	5	2	0.456	1.908(0.349,10.423)	4	3	0.448	0.551(0.118,2.573)
CA+AA	105	10	0.310	0.677(0.319,1.437)	89	26	0.046	1.723(1.009,2.941)	54	61	0.290	0.791(0.513,1.221)
rs34841297												
AA	37	1		1	30	8		1	16	22		1
A-	190	18	0.266	3.263(0.405,26.271)	171	37	0.883	0.936(0.386,2.266)	87	121	0.782	0.903(0.437,1.866)
--	191	30	0.070	6.725(0.855,5.887)	189	32	0.468	0.721(0.298,1.744)	92	129	0.724	0.879(0.429,1.799)
A-+--	381	48	0.127	4.908(0.636,37.867)	360	69	0.637	0.816(0.351,1.896)	179	250	0.741	0.890(0.445,1.779)
rs12546233												
AA	221	32		1	218	35		1	102	151		1
AC	166	16	0.080	0.554(0.285,1.074)	144	38	0.037	1.738(1.033,2.921)	82	100	0.339	0.823(0.552,1.228)
CC	31	1	0.115	0.190(0.024,1.495)	28	4	0.716	0.812(0.265,2.493)	11	21	0.306	1.511(0.686,3.329)
AC+CC	197	17	0.034	0.497(0.260,0.949)	172	42	0.081	1.563(0.946,2.580)	93	121	0.603	0.904(0.618,1.323)

[a] *P* values adjusted for age, menarche age, menopausal status, number of pregnancies, number of abortions, history of breast feeding, and family history of breast cancer in first-degree relatives in logistic regression analysis.

Supplementary Table 6. False positive report probability analysis.

Genotype		Stratification factors		*OR (95%CI)*	*P*	Priori probability			
						0.25	0.1	0.01	0.001
rs3802201	CC/CG+GG	All sample	BC	0.756(0.580,0.986)	0.039	0.124	0.299	0.824	0.979
		No family history	BC	0.717(0.544,0.944)	0.018	0.071	0.186	0.716	0.962
	CC/GG	All cases	PR	2.424(1.006,5.837)	0.048	0.505	0.753	0.971	0.997
	CC/CG+GG	All cases	PR	1.458(1.001,2.124)	0.049	0.210	0.444	0.898	0.989
	CC/GG	All cases	luminal	2.730(1.127,6.609)	0.026	0.458	0.717	0.965	0.996
	CC/CG+GG	All cases	luminal	1.478(1.009,2.146)	0.045	0.184	0.404	0.882	0.987
rs2553716	AA/AC	All sample	BC	0.739(0.560,0.973)	0.031	0.108	0.267	0.801	0.976
	AA/ AC+CC	All sample	BC	0.737(0.565,0.931)	0.024	0.038	0.105	0.565	0.929
	AA+CC/AC	All sample	BC	0.770(0.590,1.006)	0.055	0.163	0.368	0.865	0.985
	AA/ AC+CC	Age at menarche≥14	BC	0.686(0.488,0.965)	0.031	0.139	0.326	0.842	0.982
		Gravidity≥3	BC	0.702(0.495,0.994)	0.046	0.184	0.403	0.881	0.987
		No breast feeding	BC	1.959(1.072,3.581)	0.029	0.310	0.574	0.937	0.993
		No family history	BC	0.691(0.524,0.910)	0.009	0.041	0.113	0.584	0.934
	AA/ AC+CC	All cases	PR	1.457(1.000,2.124)	0.050	0.212	0.447	0.899	0.989
	AA/AC	All cases	luminal	2.233(0.985,5.061)	0.054	0.489	0.742	0.969	0.997
	AA/ AC+CC	All cases	luminal	1.526(1.041,2.237)	0.030	0.164	0.370	0.866	0.985
rs4259395	AA/AG+GG	All sample	BC	0.756(0.573,0.999)	0.049	0.154	0.353	0.857	0.984
		Age≥50	BC	0.622(0.395,0.979)	0.040	0.240	0.486	0.912	0.991
		No family history	BC	0.738(0.554,0.985)	0.039	0.135	0.318	0.837	0.981
	AA/GG	All cases	PR	1.905(1.012,3.585)	0.046	0.374	0.642	0.952	0.995
	AA/AG	All cases	luminal	2.006(1.065,3.778)	0.031	0.337	0.604	0.944	0.994
rs2588297	GG/GT	All sample	BC	0.597(0.448,0.794)	0.000	0.005	0.016	0.148	0.636
	GG/ GT+TT	All sample	BC	0.606(0.459,0.798)	0.000	0.004	0.013	0.126	0.592
	GG+TT/GT	All sample	BC	0.608(0.458,0.807)	0.001	0.007	0.019	0.178	0.686
	GG/ GT+TT	Age<50	BC	0.671(0.468,0.963)	0.030	0.151	0.348	0.854	0.983
		Age≥50	BC	0.583(0.373,0.911)	0.018	0.161	0.366	0.864	0.985
		Age at menarche≥14	BC	0.580(0.407,0.828)	0.003	0.035	0.099	0.547	0.924
		Un-menopause	BC	0.583(0.403,0.844)	0.004	0.051	0.138	0.638	0.947
		Gravidity<3	BC	0.597(0.397,0.912)	0.017	0.144	0.335	0.847	0.982
		Gravidity≥3	BC	0.638(0.446,0.912)	0.014	0.092	0.233	0.770	0.971
		Age at menopause≥45	BC	0.599(0.376,0.953)	0.031	0.219	0.458	0.903	0.989
		No history of abortion	BC	0.468(0.296,0.739)	0.001	0.050	0.135	0.633	0.946
		No breast feeding	BC	0.515(0.271,0.978)	0.043	0.372	0.640	0.951	0.995
		Have breast feeding	BC	0.623(0.457,0.850)	0.003	0.025	0.071	0.456	0.894
		No family history	BC	0.551(0.412,0.737)	0.000	0.002	0.005	0.055	0.372
	GG/TT	All case	luminal	3.716(0.987,13.996)	0.052	0.636	0.840	0.983	0.998
rs10957736	CC/TT	All sample	BC	0.562(0.348,0.907)	0.018	0.185	0.405	0.882	0.987
	CC/ CT+TT	All sample	BC	0.756(0.579,0.986)	0.039	0.124	0.299	0.824	0.979
	CC+CT/TT	All sample	BC	0.627(0.397,0.991)	0.046	0.257	0.509	0.919	0.991
	CC/ CT+TT	Age≥50	BC	0.605(0.392,0.932)	0.023	0.171	0.382	0.872	0.986
		Age at menarche≥14	BC	0.704(0.500,0.990)	0.043	0.174	0.387	0.874	0.986
		Age at menopause≥45	BC	0.633(0.404,0.992)	0.046	0.252	0.502	0.917	0.991
		No history of abortion	BC	0.619(0.398,0.961)	0.032	0.209	0.442	0.897	0.989
		No family history	BC	0.751(0.570,0.991)	0.043	0.139	0.326	0.842	0.982
rs269183	TT/ CT+CC	All cases	HER-2	1.755(1.030,2.991)	0.039	0.291	0.552	0.931	0.993
	TT/CT	All cases	Her-2 over-expression	1.802(1.031,3.150)	0.039	0.309	0.573	0.937	0.993
	TT/ CT+CC	All cases	Her-2 over-expression	1.787(1.031,3.097)	0.039	0.303	0.566	0.935	0.993
rs269198	CC/CA+AA	All cases	Her-2 over-expression	1.723(1.009,2.941)	0.046	0.312	0.576	0.937	0.993
rs34841297	AA/--	All sample	BC	1.936(1.208,3.123)	0.006	0.121	0.292	0.819	0.979
	AA/ A-+--	All sample	BC	1.704(1.087,2.672)	0.020	0.173	0.386	0.874	0.986

	AA+A-/--	All sample	BC	1.388(1.062,1.812)	0.016	0.063	0.167	0.688	0.957
	AA/ A-+--	Age<50	BC	1.998(1.072,3.723)	0.029	0.324	0.590	0.941	0.994
		Age at menarche<14	BC	2.823(1.316,6.055)	0.008	0.306	0.570	0.936	0.993
		Un-menopause	BC	2.490(1.288,4.815)	0.007	0.233	0.477	0.910	0.990
		Abortion history	BC	2.045(1.117,3.744)	0.020	0.280	0.538	0.928	0.992
		No breast feeding	BC	3.290(1.142,9.476)	0.027	0.530	0.772	0.974	0.997
		had family history	BC	1.905(1.183,3.070)	0.008	0.130	0.309	0.831	0.980
rs12546233	AA/AC	All sample	BC	0.764(0.578,1.006)	0.055	0.166	0.373	0.868	0.985
	AA/ AC+CC	All sample	BC	0.747(0.573,0.973)	0.031	0.103	0.256	0.791	0.974
		Age at menarche≥14	BC	0.699(0.496,0.984)	0.040	0.166	0.373	0.867	0.985
		No history of abortion	BC	0.517(0.333,0.804)	0.003	0.073	0.191	0.723	0.963
		No family history	BC	0.737(0.560,0.971)	0.030	0.106	0.262	0.796	0.975
		All cases	Triple-Negative	0.497(0.260,0.949)	0.034	0.354	0.622	0.948	0.955

Supplementary Table 7. Haplotype analysis of nine SNPs in *MIR2052HG.*

Gene	Haplotype[a]	Cases (%)	Controls (%)	χ^2	*P*	*OR* (95%*CI*)
MIR2052HG	C A A G C T C M[b] A	579.61(57.5)	545.74(54.0)	3.879	0.049	1.203 (1.001,1.445)
	C A A G T C A W C	14.72(1.5)	20.16(2.0)	0.787	0.375	0.737 (0.374,1.451)
	C A A T T T C W C	10.79(1.1)	6.42(0.6)	1.181	0.277	1.712 (0.642,4.565)
	C A G G T C A W A	32.98(3.3)	30.66(3.0)	0.126	0.722	1.095 (0.664,1.805)
	C A G G T C A W C	54.55(5.4)	55.25(5.5)	0.000	0.993	1.002 (0.681,1.473)
	G C G G C T C M A	90.02(8.9)	82.97(8.2)	0.440	0.507	1.112 (0.813,1.520)
	G C G T C T C M A	15.05(1.5)	10.51(1.0)	0.886	0.346	1.461 (0.661,3.228)
	G C G T T T C W A	3.06(0.3)	11.93(1.2)	5.160	0.023	0.258 (0.073,0.908)
	G C G T T T C W C	138.71(13.8)	189.63(18.8)	8.678	0.003	0.698 (0.549,0.887)

[a]The sequence of SNP locus is rs3802201; rs2553716; rs4259395; rs2588297; rs10957736; rs269183; rs269198; rs34841297 and rs12546233

[b]M represents A genotype, W represents deletion

Supplementary Table 8. Sequence of miR-4456 primers of qRT-PCR in MDA-MB-231 and MCF-10A cells.

Gene	Sequence of primers (5'-3')
miR-4456	F: ATATATCGCGCCTGGTGGCTT
	R: AGTGCAGGGTCCGAGGTATT
	RT: GTCGTATCCAGTGCAGGGTCCGAGGTATTCGCACTGGATACGACAAAAGG GCACTGGATACGACCAAGCC
U6	F: CGCAAATTCGTGAAGCGTTC
	R: GCAGGGTCCGAGGTATTC
	RT: GTCGTATCCAGTGCAGGGTCCGAGGTATTCG
